# Insights from Koala–Cattle Interaction Experiments: Koalas and Cattle May See Each Other as a Disturbance

**DOI:** 10.3390/ani12070872

**Published:** 2022-03-30

**Authors:** Alex Zijian Jiang, Andrew Tribe, Clive J. C. Phillips, Peter J. Murray

**Affiliations:** 1 School of Veterinary Science, The University of Queensland, Gatton 4343, Australia; zijian.jiang@uqconnect.edu.au; 2 Turner Family Foundation, Hidden Vale Wildlife Centre, 617 Grandchester Mount Mort Rd., Grandchester 4340, Australia; andrew.tribe@turnerfamilyfoundation.com.au; 3 Institute of Veterinary Medicine and Animal Science, Estonia University of Life Sciences, 51014 Tartu, Estonia; clive.phillips@curtin.edu.au; 4 Curtin University Sustainability Policy Institute, Perth 6845, Australia; 5 School of Agriculture and Environmental Science, University of Southern Queensland, Darling Heights 4350, Australia

**Keywords:** *Phascolarctos cinereus*, trauma, injury, mortality, cow, bull, attack, trample, risk, domestic animal, wildlife conservation

## Abstract

**Simple Summary:**

Koalas have been listed as endangered because of their rapidly declining populations. As an iconic Australian animal, effective conservation is critical to reverse the decline, which requires a comprehensive understanding of threats to koalas, such as predation, disease and habitat loss. In the last decade, livestock were suspected to be a new potential threat to koalas, with anecdotal evidence of koalas being trampled to death by livestock, especially cattle. We investigated the significance of cattle being a threat to koalas in two experiments testing how koalas and cattle perceive and react to each other when sharing space. In the first experiment, we recorded the behaviour of free-ranging koalas prior to, during and after cattle grazing within the koalas’ home ranges. Koalas showed decreased moving distance and home range size when cattle grazed in their living environment. In the second experiment, we recorded the cattle reactions to a moving koala model on a vehicle, a dog model on the same vehicle and the vehicle alone. The koala and dog models elicited similar aggression and fear in cattle, both significantly more than the vehicle alone did. The results provide confirmatory evidence of negative koala–cattle interactions and indicate that cattle and koalas may see each other as a disturbance.

**Abstract:**

Koalas are facing many threats and have now been officially listed as endangered. Recently, concerns were raised in anecdotal reports of koalas being killed by livestock, especially cattle. We investigated the significance of cattle as a threat to koala survival via two koala–cattle interaction experiments, from both the koala and cattle perspectives. In the first experiment, we recorded the ranging behaviour of free-ranging, radio-collared koalas prior to, during and after cattle grazed within their usual home range. Koalas decreased their distance travelled and the size of their home range when they shared space with cattle, compared with the period before cattle started grazing within their home range. In the second experiment, we recorded the reactions of cattle towards koalas that they encountered on the ground, using motorised animal models: a model koala mounted on a remote-controlled vehicle and a model dog mounted on the same vehicle, and the vehicle alone. The koala model elicited aggression and fear in cattle, similar to the dog model, whereas their reaction to the vehicle was significantly less aggressive. No actual attacks by the cattle were observed. The results provide experimental evidence that negative koala–livestock interactions occur and indicate that cattle and koalas may see each other as a disturbance.

## 1. Introduction

As an iconic Australian species, the koala (*Phascolarctos cinereus*) has received considerable attention from both the general public and scientists over the past century. Despite this, there is an ongoing decline of koala populations [1,2]. In 2012, the koala was listed as “Vulnerable to extinction” in Queensland and also by the International Union for Conservation of Nature (IUCN) Red List in 2016 [3]. However, in February 2022, the federal conservation status of koalas was downgraded to “Endangered” in Queensland, New South Wales and the Australian Capital Territory [4]. Habitat loss and fragmentation [5,6] are considered to be major threats to koala survival, as are disease (especially chlamydiosis) [7], dog predation and attack [8], vehicle strike [9], climate change [10] and bush fires [11].

In Queensland, the clearing of koala habitats in rural inland areas is mainly associated with the expansion of cattle grazing from the beef industry [12], leading to large areas where koala habitats and livestock grazing overlap. Recently, management strategies were proposed (W. Ellis, pers. comm., 10 October 2017) to facilitate the conservation of koalas on cattle grazing land. These strategies encourage the preservation of remnant koala habitats on grazing properties with vegetation corridors to connect these remnant habitats so that resident koalas are able to survive and safely traverse to new areas, while maintaining the profitability and sustainability of the livestock property.

The success of koala conservation on livestock grazing land requires in-depth knowledge about the viability of their co-existence, and especially any potential for conflicts between koalas and livestock. Previous research on wildlife–livestock conflicts has focused on livestock as victims of predation from large carnivores [13,14,15] and even attacks from elephants [16]. Elsewhere, livestock gradually exclude resident large mammalian herbivores from food, water and space in rangelands [17,18,19,20,21,22]. The extensive grazing of livestock has also been associated with damage to ground vegetation, which may lead to a decline of terrestrial fauna species adapted to dense ground cover [23,24]. However, some scientists believe that arboreal species may not be negatively impacted by grazing cattle in the same landscape [25,26,27,28].

Koalas, as general arboreal animals, usually only come down to the ground when moving from tree to tree [29], but little is known about interactions between koalas and livestock where they co-exist. In the last decade, there have been anecdotal reports that koalas were trampled to death by livestock, especially cattle, in paddocks where grazing livestock and koalas overlap [30,31,32,33,34]. In 2021, Jiang et al. [35] reported evidence of livestock-inflicted harm to koalas, obtained from an online survey and analyses of databases from wildlife hospitals in Queensland and a wildlife rescue group in Victoria. The proportion of livestock–koala incidents among all koala admissions recorded was relatively low (less than 0.8%) but increasing, and was also believed to be greatly under-reported. Meanwhile, the death rate of koalas from livestock attacks was reported to be high (75%, similar to the koala death rate from vehicle strikes (80.3%) and exceeding that from chlamydiosis (50%) [9,36,37,38]. Among livestock, cattle were reported to be responsible for the majority (75%–100%) of livestock–koala incidents [35]. Other than koala mortalities, there have been ongoing reports of deaths and injuries to farm workers [39] or walkers passing through paddocks with grazing cattle [40]. It is likely, therefore, that livestock, especially cattle, are a threat to koalas within the areas where they co-exist.

Although the incidents of livestock attacking koalas were confirmed by witness reports and clinical examinations [35], the circumstances behind these attacks are unclear. The koala–livestock relationship is particularly complicated when they have been anecdotally observed to be sharing the same space with no conflicts, and koalas often use livestock grazing land as part of their home range [41,42]. This observed co-existence indicates that their encounters may not always be agonistic, and koalas may not perceive livestock as a major threat.

To test interactions between two species, particularly where aggression may result in the injury or death of one of the species, realistic models have been widely used between different species [43,44,45]. To test the responses of cattle to an animal that they may fear or show aggression to, it is necessary to test them in herds, as cattle are social animals, likely to only display normal behaviour when in herds. In contrast, koalas are solitary animals, except during the breeding season and, thus, to test their normal behavioural responses to cattle, it is necessary to test them individually. This study assessed interactions between cattle and koalas using two experiments. Experiment 1 tested the reactions of free-ranging koalas towards grazing cattle in their natural habitat, with a hypothesis that koalas would have inhibited ranging behaviour or show avoidance of grazing cattle. Experiment 2 tested the reactions of cattle of different age–sex classes towards koalas encountered on the ground, using a motorised koala model due to ethical concerns surrounding using real koalas for tests, and the technical difficulties in observing natural koala–livestock encounters in the field. Our hypothesis was that cattle would show aggressive behaviour towards the koala model, and cows in the calving season would show more aggression.

## 2. Materials and Methods

### 2.1. Study Site

This study was conducted on an Old Hidden Vale (OHV) property which is 7 km south of Grandchester in south-east Queensland. The 5000 ha property consists of 70% remnant eucalypt forest as high-quality koala habitat and 30% cleared land used for beef cattle grazing.

### 2.2. Experiment 1: Koala Reactions towards Cattle

Experiment 1 was conducted in 25 ha of remnant eucalypt forest known to have a high koala population, the “Koala Hot Spot”. Dominated by *Eucalyptus tereticornis* and *E. crebra*, the OHV Koala Hot Spot had the highest density of koalas across OHV, and was also periodically used for cattle grazing. In 2018, in the Koala Hot Spot, four male and five female koalas were captured and fitted with tracking collars (LX telemetry monitoring system (LX GROUP™)). The LX collars reported the Global Positioning System (GPS) locations of these koalas on a 12-hourly basis (at 10:00 and 22:00 daily).

**Figure 1:**
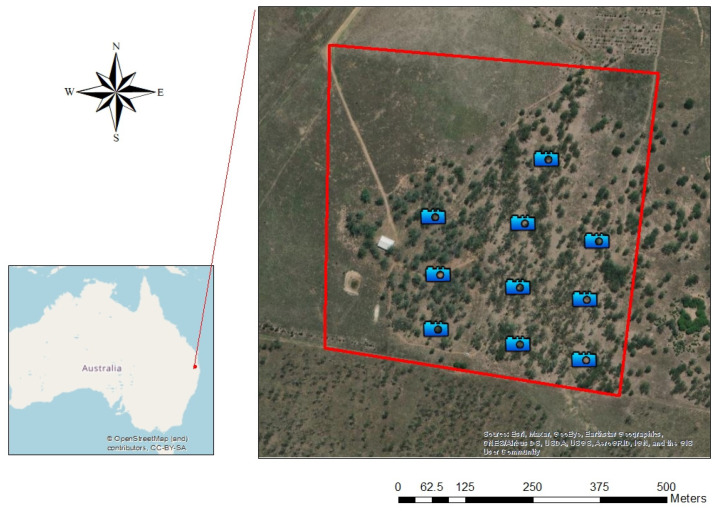
Old Hidden Vale Koala Hot Spot, with 10 cameras evenly distributed within the area with trees.

Grazing cattle herds (Droughtmaster and Charolais cross-cattle, four to ten years old) were put into the OHV Koala Hot Spot four times, recorded as four tests, during 2019–2021. Table 1 shows the dates, number of days and animals used in each test. To record the locations of the cattle during tests, we distributed 10 cameras (Enduro™ Swift) within the area with trees (Figure 1). Koala home range size (calculated using three methods: Minimal Convex Polygon (MCP) [46], 90% Kernel Utilisation Distribution (KUD) and 50% KUD [47,48,49]) and 12-hourly travel distance were calculated for each test period. Similar data were also collected from the periods before and after each test, both of which were of the same length as the matching test. For example, Test 1 lasted eight days; hence, the before and after Test 1 periods were set to cover the eight days immediately before and after Test 1. The home range sizes of koalas were calculated within R using the adehabitatHR library of functions [50] via the Zoatrack online platform [51]. We used linear mixed models, with individual koala, test and their interaction as random effects, to test whether koala home range and travel distance varied before, during and after periods when cattle grazed in the Koala Hot Spot.

### 2.3. Experiment 2: Cattle Reactions towards a Koala Model

**Figure 2:**
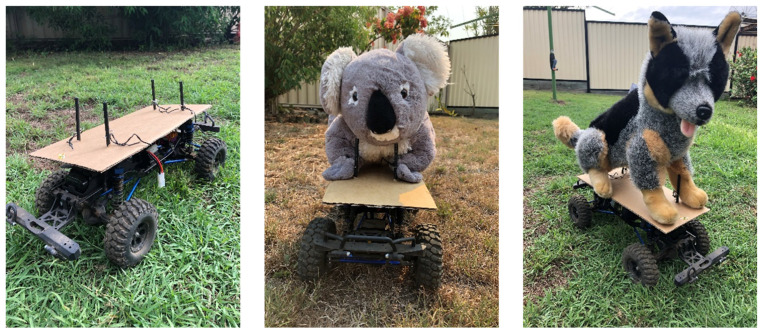
The three treatments (from left to right): Car, Koala and Dog, used to test the response of cattle to koala and dog models.

We tested the reactions of herds of cattle grazing in a paddock to three types of treatments: a radio-controlled vehicle (Car), a moving koala model (Koala) and a moving dog model (Dog) (Figure 2). The radio-controlled vehicle (length 50, width 18, height 18 cm) was used to determine whether the motor noise, vehicle wheel movement and smell may have influenced cattle behaviour. In this way, any differences in the cattle reactions towards the Car and stimuli (Koala or Dog) could be identified as the reactions of cattle towards the koala/dog model itself without the influences from the radio-controlled vehicle. The koala model was a life-sized (length 35, width 22, height 25 cm) koala toy mounted on the radio-controlled vehicle to simulate a koala moving on the ground. Similarly, the dog model was a life-sized dog toy (length 50, width 20, height 42 cm) mounted on the radio-controlled vehicle to simulate a dog moving on the ground (Figure 2). Due to practical concerns of balance and stability, we used a half sitting dog model, which may have possibly led to a lessened reaction by the cattle, as a sitting dog may be considered less threatening than a standing dog. The purpose of the dog model was to test whether cattle reacted to both the koala and dog models, indicating that cattle would react to koalas in the same way as to dogs. The koala and dog models were sprayed with koala and dog urine and faeces, respectively, to give the models the scent of real animals.

The cattle (Droughtmaster and Charolais crosses, four to ten years old) were categorised into five classes: (1) non-lactating cows (NLC)—single cows which had previously bred, (2) lactating cows with old calves (LCO)—cows with dependent calves that were between eight and ten months old, (3) lactating cows with young calves (LCY)—cows with dependent calves that were less than three months old, (4) heifers (HF)—young adult cows about one year old without any breeding history, and (5) adult bulls (BL). The numbers of cattle herds tested in each class and the numbers of animals in each herd are shown in Table 2. Calves were dependent on their mothers, so the two lactating cow classes had 20 animals (10 cows and 10 calves) in each herd. As only seven adult bulls were available on the OHV property (for breeding purposes) they were divided into three herds with two, two and three bulls in each herd.

The three treatments, i.e., Car, Koala and Dog, were tested on each cattle herd within a single day at 09:00, 12:00 and 15:00. In order to reduce sequence bias, in each cattle class (except bulls), the sequence of the three treatments across the six herds was rotated as per the Balanced Latin Square design shown in Table 3. Bulls had three herds only, which formed a Latin Square design for the first three herds in Table 3.

**Figure 3:**
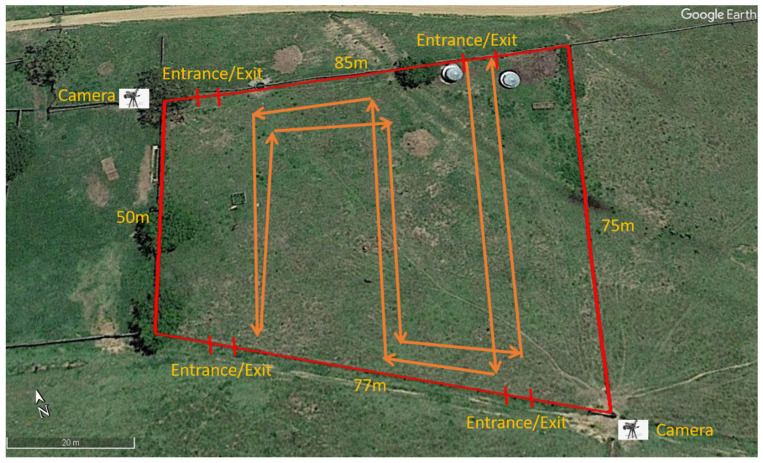
Paddock used for koala–cattle tests (red lines as the boundary) showing an example of the vehicle/model travel route (arrowed orange lines) from one of the four possible entrance/exit points. Map generated with GoogleEarth™.

On each test day, the relevant herd was put into a 0.5 ha paddock (Figure 3) at 07:00 and removed at 17:00 after the afternoon test. During each test, the motorised model entered and exited the paddock from the same point, with a standard predetermined route shown in Figure 3. There were four options for the entrance/exit point to the paddock. For each test, the entrance/exit point was selected prior to the test based on the cattle location: the point with the greatest distance to the herd was chosen so that the cattle had similar time to assess and react to the stimulus before it moved close to them. The vehicle was operated by a researcher hiding behind an obstacle (i.e., a car) outside of the paddock to minimise any distraction to the cattle. The maximum speed of the vehicle was 1.3 m/s, which was within the koala moving speed range when on the ground [52].

All tests were recorded by two cameras (GoPro™ Hero7) placed at diagonally opposite corners of the paddock (Figure 3). The cattle’s behavioural reactions towards the treatments were coded and extracted with the aid of the software programme BORIS™ (Behavioural Observation Research Interactive Software) [53]. The complete list of cattle behaviours is included in Appendix A. In the data analyses, we focused on the cattle reactions shown in Table 4, as other behaviours, such as attack and sniff, were absent or extremely rare.

In the data analyses, we focused on herd behaviour measurements and used herds as sample units. This was because individual behaviours were not independent of each other as they are herd animals, and thus their behaviour can be affected by others in the herd. For binomial behaviours (i.e., threat display, herding and approach), the herd was marked “Yes” if any of the animals in that herd displayed the behaviour in each test. Because the binomial result of a herd can be biased by the varying animal numbers (i.e., there is a higher chance to observe a specific behaviour when the animal number is high), the statistical models for these binomial variables were weighted by the reciprocal of cattle number in each herd. However, given the extremely low number of bulls (between two and three) in each herd, bulls were excluded in binomial models (i.e., threat display, herding and approach behaviours). For avoidance and other-type behaviours (but not recovery), which were measured by duration, we focused on the aggregated amount of the behaviour displayed by all the animals in the herd during a test. There were variations of test duration and the number of cattle used in each test. Therefore, in each test, the herd measurements of avoidance and other-type behaviours were rescaled to every cattle second (i.e., aggregated behaviour duration/(test duration x total number of animals in the test)) to adjust for the variation in test duration and number of animals, denoted as adjusted proportion (Table 4). Provided that each cattle herd had undergone all three treatments, we used linear mixed models (for quantitative analysis) and generalised linear mixed models (for binomial analysis), with herd as a random effect, to test whether cattle of various classes had different levels of reactions towards the Car, Koala and Dog treatments. The modelling was implemented in R version 3.5.1 [54] using the lme4 [55] and lmerTest [56] libraries of functions.

## 3. Results

### 3.1. Experiment 1: Koala Reactions towards Cattle

Cattle were recorded grazing by all 10 cameras in the OHV Koala Hot Spot on a daily basis, and thus we assumed that all koalas were aware of the cattle grazing within their home range during tests. No koalas were injured or killed by cattle during the tests. There were significant effects of cattle grazing (i.e., between periods before, during and after cattle grazing) on koala 12-hourly travel distance and home range size (MCP, 90%KUD and 50%KUD). The koalas showed the shortest travel distance and smallest home range size during the cattle grazing period, which were significantly lower than the period before cattle grazing. After cattle grazing, koalas showed increased travel distance and home range size, which was not significantly different from the period during and before cattle grazing (Table 5).

### 3.2. Experiment 2: Cattle Reactions towards a Koala Model

Although no actual attacks involving physical contact to the treatments were observed from cattle, cattle showed aggression in the form of threat displays, including ground pawing, head swing and charging towards the treatments in this study. There were significant effects of treatment (Car, Koala and Dog in Table 6) and cattle class (lactating cows with old calves, lactating cows with young calves, heifers, dry cows and bulls in Table 7) on various cattle behavioural reactions towards treatments. No interactions between the effects of treatment and cattle class were detected.

In relation to the treatment effect, the dog and koala models elicited similar, and both significantly higher, probabilities of cattle showing threat display and herding behaviour than the vehicle did. Cattle took a significantly longer time to recover and disengage from the dog and koala models after the test was completed, compared with the vehicle. The vehicle elicited more other-type behaviours in cattle than the dog model, but not the koala model. The cattle had similar minimum flight distances when facing the dog and koala models, both greater than when facing the vehicle. The vehicle elicited a higher probability of cattle showing approach behaviour than the koala and dog models did. Cattle avoidance behaviours were not affected (Table 6).

In relation to the cattle class effect, lactating cows with old calves (LCO) had a higher probability of showing herding behaviour than all other classes. LCO and bulls (BL) had the highest recovery time taken to disengage after the test, with LCO, but not BL, greater than non-lactating cows (NLC); both LCO and BL were greater than lactating cows with young calves (LCY) and heifers (HF) and NLC. LCO and LCY had the highest probability of showing threat displays, both higher than HF and NLC, with HF higher than NLC. LCO showed fewer other-type behaviours than any other cattle classes, except NLC; HF exhibited more than any other classes. Avoidance and approach behaviours and minimum flight distance were not affected by cattle class (Table 7). Although bulls were excluded from binomial analyses of threat display, herding and approach behaviours, they showed none of these behaviours.

## 4. Discussion

Experiment 1 facilitated our understanding of koala–cattle interactions from the perspective of koalas. Grazing cattle may act as a source of disturbance leading to a decrease in koalas’ other activities and ranging behaviour. This was reflected by the decreased 12-hourly travel distance and home range size of all koalas when sharing space with cattle, compared with the period before cattle grazing started. In particular, the trend of koala ranging behaviour in the period before, during and after cattle grazing showed great consistency. The travel distance and all types of home range sizes of koalas were the greatest before cattle arrived, and they significantly dropped to the lowest level during cattle grazing and started to recover after cattle were removed from the study site.

Koalas are tree dwellers that come to the ground when moving from one tree to another. Despite the uncertainty of the actual time that koalas stay on the ground, the decrease in koala ranging behaviour observed in this study indicated that koalas might be minimising their time staying on the ground, and hence the chance of encountering any cattle grazing within their home range. Once the cattle were removed, koalas could be more relaxed with their ranging behaviour, which gradually recovered back to the level before cattle were placed in their environment. However, this study observed no koalas deserting or moving away from their usual home range to avoid cattle. Other research also reported the stable co-existence of koalas and livestock in paddocks that contain koala fodder trees [35,41,42]. Therefore, we suspect that koalas see cattle as a disturbance that has a limited impact on their home site choice. Lastly, the potential occurrence of agonistic koala–cattle encounters could have been missed in this experiment, as we were unable to monitor the koalas constantly. Although none of the koalas were injured or killed by cattle during the experiment, the possibility of cattle attempting to chase and attack koalas encountered on the ground could not be excluded.

Experiment 2 indicated that the koala and dog models triggered higher levels of aggression and fear in the cattle than the car did. This was reflected by cattle having:

(1) A greater likelihood of threat display towards the koala and dog models. Threat displays of animals involve ritualised aggression observed in conflict situations when confronting a threat, which can be either a conspecific competitor or a predator. Other than physical attacks, a threat display is a vital indicator of animals’ aggression and often leads to an escalation from non-contact to contact agonistic behaviour [57,58,59]. In this study, when confronted with the dog and koala models, cattle were more aggressive by being more inclined to display behaviours such as ground pawing, head swinging and charging. These behaviours of cattle have been often observed when confronted with a threat (e.g., predator) [60,61], and thus it may suggest that cattle saw the koala and dog models as threats.
(2) A greater likelihood of herding behaviour when facing koala and dog models. This is recognised as gregarious behaviour, which is a typical collective behaviour observed in many insects, fish and mammals [62,63]. It is driven by fear of predators, with individuals responding to potential danger by forming a firm group and animals moving towards its centre [64,65]. The greater occurrence of cattle herding behaviour could be associated with a higher level of fear caused by the dog and koala models, which could be perceived by the cattle to be predators.
(3) Longer time to recover back to other-type behaviours after koala and dog model tests. This indicated a higher level of distress [66] as cattle needed a longer time to disengage themselves from the model treatments.
(4) A tendency for fewer other-type behaviours when tested with koala and dog models, although the difference between the koala and vehicle was not significant but showed a similar trend. Other-type behaviours represent relaxation and disengagement from the treatment; on the contrary, animals increase vigilance at the expense of normal (other-type) behaviour in response to threats. Therefore, vigilance is believed to be an indicator of fear in animal behaviour research [67,68]. In this study, the longer period of other-type behaviours when facing the car indicates that cattle were more relaxed when without the dog or koala models. On the other hand, the decrease in other-type behaviours reflected an increase in vigilance in cattle, i.e., fear and stress, especially when confronted with dog models.
(5) Greater minimum flight distance when approached by the dog and koala models. Flight distance indicates the alertness of an animal towards a stimulus and is widely used to measure fear [69,70,71]. In this study, cattle allowed the car to approach closer than the dog and koala models before avoidance, suggesting that cattle were more cautious and fearful when seeing koala or dog models.
(6) A greater likelihood of approach towards the car. The approach could be an exploratory behaviour that suits the inquisitive nature of cattle [60], indicating cattle were more relaxed with the vehicle by showing their natural behaviour. The approach could also be a confronting and courageous behaviour towards a possible threat, indicating that cattle were less threatened when without the dog and koala models.

Undoubtedly, neither real koalas nor dogs walk like the models moved in this study. However, provided that the car took into account the confounding attributes (i.e., scent, noise, shape and material) from the vehicle, the different cattle reactions to Koala and Dog represent the cattle’s reaction towards the koala/dog model itself. Our results supported the assumption that cattle are behaviourally responsive towards animal models. Previous research showed that a live dog triggered higher levels of fear in domestic sheep than taxidermic models did [72]. Another study reported that a large portion of the repertoire of minnow anti-predator behaviours, which were often observed with live predators, was absent when using predator models as stimuli [66]. Therefore, although only threat displays of cattle were observed in this study, more aggressive reactions, i.e., physical attacks, towards live koalas in the field are likely.

Dogs naturally present as a predator threat to cattle. Studies suggested that domestic and wild ungulates show less normal behaviours and higher vigilance with the presence of predator stimuli [45,73,74,75]. Our observation matched this natural instinct of cattle by showing that the dog model had triggered the highest level of fear and stress related reactions of cattle, e.g., the highest probabilities of threat display and herding behaviour and the shortest period of other-type behaviours. Meanwhile, the post hoc analyses of cattle reactions showed a consistent pattern of similarity between the dog and koala model effects, both of which were significantly different from the car effect. It could be evidence that cattle may treat koalas as a similar threat, or at least a disturbance, to dogs. Alternatively, it could be possible that the expected higher level of cattle reactions towards the dog model was offset by its sitting pose, which may have presented a relaxed dog and, hence, elicited fewer cattle reactions. Nevertheless, we cannot exclude the possibility that cattle did not relate the models to real animals and were not interested in the differences between the two animal models in this study (i.e., two furry objects with different scents and shapes), and thus reacted in a similar way. If so, the elicited cattle reactions were due to the strangeness of the moving visual stimuli instead of reacting to real animals. Similarly, the different sizes between the models and the vehicle (i.e., koala and dog models were of a similar size, and both were larger than the car alone) may have contributed to the lower level of cattle reactions towards the vehicle. Future studies are required to investigate the likelihood of cattle perceiving the models as live animals.

Lactating cows with calves were more aggressive than dry cows, heifers and bulls. This was reflected in the cattle class effects on cattle reactions, whereby both lactating cow classes (with young and old calves) had greater probabilities to show threat display towards stimuli, which can be explained by the maternal drive of mother cows [39,40,76]. This observation also supports the findings of Jiang et al.’s survey of livestock–koala incidents [35], where cows with calves at foot are more likely to attack koalas when encountered on the ground. However, the two classes of lactating cows showed a deviation in fear-based responses, including herding, recovery and other behaviour. In particular, lactating cows with old calves spent a significantly longer time on recovery, less time on other-type behaviour and were more likely to show herding behaviour than lactating cows with young calves. A possible explanation could be the sensitive period of calf development [76]; young calves less than three months old may not be fully mentally developed regarding the cognition of potential threats in living environment, and thus tend to be less fearful with more other-type behaviour, less herding and showing quicker recovery. On the other hand, older calves of between eight and 10 months old could be more aware of potential threats and showed more fear towards stimuli.

## 5. Conclusions

The objective of this study was to investigate the relationship between free-ranging koalas and grazing cattle from the perspectives of both cattle and koalas. For cattle, the artificial koala model elicited aggression and fear similar to the dog model. Although no physical attacks from cattle towards the stimuli were observed in this study, it is possible that the cattle would have been more aggressive towards live animals rather than models. For koalas, the presence of grazing cattle within their home range reduced their travel distance and home range size. Therefore, koalas and cattle could see each other as a stressor or disturbance. Along with the existence of koala mortalities caused by cattle, which was investigated in our previous research, we suspect that cattle are acting as a low-risk threat to koalas in the areas they overlap. The results provide new information about interactions between koalas and cattle in rural regions of Australia. This study was limited by the use of the artificial models due to ethical and practical concerns, and future studies investigating how likely it is that cattle perceive the models as live animals are encouraged. More advanced monitoring technology, such as mini-cameras suitable to be attached on koalas and livestock, could be used to determine the tolerance of livestock towards koalas in future longer-term studies.

## Figures and Tables

**Table 1:** The dates, number of days and the number of cattle and koalas studied in the four koala reaction tests.

Test	Days	Start	End	No. of Cattle	No. of Koalas
1	8	21 February 2019	28 February 2019	130	4
2	6	23 April 2019	28 April 2019	73	5
3	12	1 May 2020	12 May 2020	81	8
4	11	2 February 2021	12 February 2021	116	5

**Table 2:** The number of herds tested in each cattle class, the number of animals in each herd, and the time when testing was undertaken. Lactating old = lactating cow with old calves, Lactating young = lactating cows with young calves.

Cattle Class	Time	Herds	Cattle Per Herd	Total Cattle
Dry cows	July 2020	6	10	60
Heifer	May 2020	6	10	60
Lactating old	March 2020	6	20	120
Lactating young	November 2020	6	20	120
Bull	August 2020	3	2, 2 and 3	7
Sum		27		367

**Table 3:** Balanced Latin Square design of treatment sequence across the six herds of each cattle class.

Time	Herd 1	Herd 2	Herd 3	Herd 4	Herd 5	Herd 6
09:00	Koala	Car	Dog	Koala	Dog	Car
12:00	Dog	Koala	Car	Car	Koala	Dog
15:00	Car	Dog	Koala	Dog	Car	Koala

**Table 4:** Description and measurements of the behaviours of cattle recorded interacting with the car, koala and dog models. Adjusted proportion = aggregated behaviour duration/(test duration × total number of animals in the test).

Behaviour	Description	Measurement as Individual	Measurement as Herd
Approach	Cattle moving towards the stimulus at normal walking speed	Binomial (Yes/No)	Binomial (Yes/No)
Avoidance	Cattle moving away or escaping from the stimulus, or joining herd	Duration (seconds)	Adjusted proportion (%)
Herding	The quick gathering of at least 80% of cattle in a test after the stimulus emerged, with a maximum of three metres between each individual and its nearest neighbour	n/a	Binomial (Yes/No)
Minimum flight distance	The minimum distance between the stimulus and any cattle in a test before the cattle moved away	n/a	Length (metres)
Other	Cattle normal behaviours which were disengaged from the stimulus, including feeding, drinking, resting, standing, walking and social behaviours	Duration (seconds)	Adjusted proportion (%)
Recovery	The time taken by at least half of the cattle in a test to start to display any “Other” behaviours after the stimulus exited the paddock	n/a	Duration (seconds)
Threat display	Non-contact ritualised aggression, including head swing, charging, pawing ground and air kick	Binomial (Yes/No)	Binomial (Yes/No)

**Table 5:** Effects of cattle grazing on koala activity levels, 12-hourly travel distances and home range sizes (HR = home range, MCP = Minimal Convex Polygon, KUD = Kernel Utilisation Distribution) in Experiment 1. Within each koala response, periods followed by the same superscript are not significantly different, using Tukey’s test.

Koala Response	Statistics and Value	*p* Value	Period	Mean	CI-Lower	CI-Upper
12-hourly travel distance (metre)	F_2.45_ = 4.839	0.013	During	31.62 ^a^	22.39	45.71
			After	37.15 ^ab^	25.70	53.70
			Before	44.67 ^b^	30.90	64.57
HR-MCP (hectare)	F_2.48_ = 3.870	0.027	During	0.76 ^a^	0.38	1.55
			After	1.00 ^ab^	0.49	2.04
			Before	1.41 ^b^	0.69	2.95
HR-90%KUD (hectare)	F_2.45_ = 4.814	0.013	During	2.40 ^a^	1.29	4.57
			After	3.31 ^ab^	1.74	6.31
			Before	5.13 ^b^	2.63	10.0
HR-50%KUD (hectare)	F_2.44_ = 5.477	0.007	During	0.74 ^a^	0.37	1.48
			After	0.97 ^ab^	0.49	1.95
			Before	1.58 ^b^	0.77	3.16

**Table 6:** Effects of treatments (Koala = koala model, Dog = dog model, Car = car without models) on cattle behavioural reactions in Experiment 2. Within each behaviour, treatments followed by the same superscript are not significantly different, using Tukey’s test. Adjusted proportion = aggregated behaviour duration/(test duration × total number of animals in the test). All percentage (%) means are within a test period.

Behaviour	Statistics and Value	*p* Value	Treatment	Mean	CI-Lower	CI-Upper
Herding (binomial probability, %)	X^2^_2_ = 6.423	0.040	Car	26.6 ^a^	9.7	55.0
			Koala	65.4 ^b^	37.9	85.5
			Dog	76.4 ^b^	48.8	91.6
Recovery (seconds)	F_2.48_ = 3.197	0.049	Car	11.6 ^a^	−2.7	26.0
			Koala	29.8 ^b^	15.7	43.9
			Dog	30.7 ^b^	16.3	45.1
Avoidance (adjusted proportion, %)	F_2.52_ = 0.919	0.405	Car	16.1 ^a^	11.3	20.9
			Dog	18.0 ^a^	13.4	22.7
			Koala	19.5 ^a^	14.8	24.3
Minimum flight distance (metres)	F_2.52_ = 6.822	0.002	Car	3.00 ^a^	2.25	3.75
			Dog	3.93 ^b^	3.17	4.68
			Koala	3.96 ^b^	3.21	4.72
Threat display (binomial probability, %)	X^2^_2_ = 39.376	<0.001	Car	17.8 ^a^	11.9	25.9
			Koala	49.0 ^b^	38.3	59.7
			Dog	58.8 ^b^	48.0	68.8
Approach (binomial probability, %)	X^2^_2_ = 46.509	<0.001	Dog	54.3 ^a^	12.0	91.2
			Koala	54.4 ^a^	12.0	91.2
			Car	98.3 ^b^	85.1	99.8
Other (adjusted proportion, %)	F_2.44_ = 2.992	0.061	Dog	31.5 ^a^	27.0	35.9
			Koala	34.1 ^ab^	29.5	38.6
			Car	37.6 ^b^	33.0	42.2

**Table 7:** Effects of cattle classes (LCY = lactating cows with young calves less than three months old, LCO = lactating cows with old calves that were between eight and ten months old, NLC = non-lactating cows, HF = heifers, BL = bulls) on cattle behavioural reactions in Experiment 2. Within each behaviour, cattle classes followed by the same superscript are not significantly different, using Tukey’s test. Adjusted proportion = aggregated behaviour duration/(test duration × total number of animals in the test). All percentage (%) means are within a test period.

Behaviour	Statistics and Value	*p* Value	Class	Mean	CI-Lower	CI-Upper
Herding (binomial probability, %)	X^2^_3_ = 8.984	0.029	LCY	21.7 ^a^	6.0	54.7
			HF	34.6 ^a^	11.9	67.5
			NLC	57.2 ^a^	26.1	83.5
			LCO	93.7 ^b^	65.9	99.1
Recovery (seconds)	F_4.21_ = 12.507	<0.001	HF	0.18 ^a^	−0.46	1.61
			LCY	0.23 ^a^	−0.44	1.71
			NLC	1.87 ^ab^	0.30	5.35
			BL	10.97 ^bc^	2.91	35.64
			LCO	34.89 ^c^	13.62	87.11
Avoidance (adjusted proportion, %)	F_4.22_ = 1.210	0.335	HF	12.4 ^a^	4.6	20.2
			BL	14.3 ^a^	3.3	25.3
			LCY	18.3 ^a^	10.5	26.0
			LCO	21.0 ^a^	13.2	28.8
			NLC	23.5 ^a^	15.6	31.2
Minimum flight distance (metres)	F_4.22_ = 1.138	0.364	BL	2.11 ^a^	0.07	4.15
			LCY	3.33 ^a^	1.89	4.78
			HF	3.67 ^a^	2.22	5.11
			LCO	3.67 ^a^	2.22	5.11
			NLC	4.61 ^a^	3.17	6.05
Threat display (binomial probability, %)	X^2^_3_ = 258.072	<0.001	NLC	4.4 ^a^	2.3	8.2
			HF	19.8 ^b^	14.4	26.3
			LCY	80.7 ^c^	70.8	87.8
			LCO	80.7 ^c^	70.8	87.8
Approach (binomial probability, %)	X^2^_3_ = 4.334	0.227	NLC	31.7 ^a^	0.7	96.6
			HF	35.3 ^a^	0.8	97.4
			LCY	87.7 ^a^	11.5	99.7
			LCO	99.5 ^a^	62.4	100
Other (adjusted proportion, %)	F_4.22_ = 18.851	<0.001	LCO	15.5 ^a^	8.3	22.8
			NLC	24.2 ^ab^	17.0	31.5
			BL	35.1 ^bc^	24.9	45.3
			LCY	39.0 ^c^	31.9	46.2
			HF	57.9 ^d^	50.6	65.2

## Data Availability

The data that support the findings of this study are available upon reasonable request from the corresponding author.

## Appendix A. Cattle Behaviour List

Approach: move towards the stimulus at normal walking speed, if not moving to join and stay with the main herd (>5 cattle), quantified by the binomial presence (Yes/No) and the duration of the behaviour.

Attack: any form of physical agonistic contact with the stimulus, including head butt, horn stab, trample, stomp and kick. These were not observed in this study.

Avoidance: quantified by the duration of the behaviours including Escaping, Joining herd and Moving away.

Escaping: run fast away from the stimulus.

Joining herd: move towards then stay with the majority (>50%) of the herd when the stimulus was in the same direction.

Moving away: walk away from the stimulus at normal walking speed.

Herding behaviour: the quick gathering of at least 80% of cattle during a test after the stimulus emerged, with a maximum of three metres between each individual and its nearest neighbour, quantified by the binomial presence (Yes/No) and the duration of the behaviour. This behaviour did not apply to bulls due to the low number of animals (2–3) in each test.

Lick/sniff: lick or sniff the stimulus in close distance, quantified by the counts of the behaviour.

Minimum flight distance: the minimum distance between the stimulus and any cattle during a test before the cattle moved away.

Other behaviour: disengaging from the stimulus, quantified by the duration of the behaviours including Drinking, Feeding, Resting, Social, Standing and Walking.

Drinking: drink water from a trough.

Feeding: graze on grass or eat hay.

Resting: lie on the ground.

Social: non-agonistic interactions with other cattle, e.g., grooming.

Standing: staying still with both head and body turning more than 90° from the stimulus for more than 10 s.

Walking: locomotion of cattle if still disengaged from the stimulus (i.e., showing any other behaviours) before and after movement.

Recovery time: the time taken by at least half of cattle in a test to disengage from the stimulus (i.e., start to display any other-type behaviours) after the test was completed.

Threat display: non-contact ritualised aggression, quantified by the binomial presence (Yes/No) and the counts of the behaviours including Air kick, Charging, Head swing and Pawing ground.

Air kick: kick legs in the air.

Charging: move fast towards then stop before engaging in contact with the stimulus.

Head swing: quickly lift the head up, then lower it down when facing the stimulus.

Pawing ground: dig or paw the ground with legs.
